# Anti-Heartbeat-Evoked Potentials Performance in Event-Related Potentials-Based Mental Workload Assessment

**DOI:** 10.3389/fphys.2021.744071

**Published:** 2021-10-18

**Authors:** Sangin Park, Jihyeon Ha, Laehyun Kim

**Affiliations:** ^1^Center for Bionics, Korea Institute of Science and Technology, Seoul, South Korea; ^2^Department of Biomedical Engineering, Hanyang University, Seoul, South Korea; ^3^Department of HY-KIST Bio-Convergence, Hanyang University, Seoul, South Korea

**Keywords:** electroencephalography, heartbeat-evoked potentials, event-related potentials, mental workload, subjective mental effort questionnaire

## Abstract

The aim of this study was to determine the effect of heartbeat-evoked potentials (HEPs) on the performance of an event-related potential (ERP)-based classification of mental workload (MWL). We produced low- and high-MWLs using a mental arithmetic task and measured the ERP response of 14 participants. ERP trials were divided into three conditions based on the effect of HEPs on ERPs: ERP_HEP_, containing the heartbeat in a period of 280–700ms in ERP epochs after the target; ERP_A-HEP_, not including the heartbeat within the same period; and ERP_T_, all trials including ERP_A-HEP_ and ERP_HEP_. We then compared MWL classification performance using the amplitude and latency of the P600 ERP among the three conditions. The ERP_A-HEP_ condition achieved an accuracy of 100% using a radial basis function-support vector machine (with 10-fold cross-validation), showing an increase of 14.3 and 28.6% in accuracy compared to ERP_T_ (85.7%) and ERP_HEP_ (71.4%), respectively. The results suggest that evoked potentials caused by heartbeat overlapped or interfered with the ERPs and weakened the ERP response to stimuli. This study reveals the effect of the evoked potentials induced by heartbeats on the performance of the MWL classification based on ERPs.

## Introduction

Event-related potentials (ERPs) provide a powerful method for interpreting the relationship between the human mind and the brain. ERPs measure brain responses which are a direct result of a specific input in the form of sensory, cognitive, memory, or motor events (Luck, 2014). Because brain activity in response to a single event or stimulus is not usually visible in electroencephalogram (EEG) signals, the ERP technique is required to measure the response to a stimulus in many trials (Coles and Rugg, 1995; Boudewyn et al., 2018). The brain activity of a single event or stimulus trial would be averaged out, and the relevant or dominant potentials would remain (Coles and Rugg, 1995).

Electroencephalogram signals are often contaminated by various artifacts, such as eyeblinks, ocular movements, and muscular and cardiac activity, which are typically not of interest (Uriguen and Garcia-Zapirain, 2015). These artifacts can affect EEG signals and interfere with relevant or dominant potentials in ERPs. Thus, many previous studies have sought to remove artifacts, such as muscular activity (Chen et al., 2019; Zou et al., 2020), cardiac activity (Hamaneh et al., 2014; Dai et al., 2019), eyeblinks, and ocular movements (Dimigen, 2020; Egambaram et al., 2020). The effect of noise on ERP analysis has been minimized thanks to the development of methods to remove noise in EEG signals. However, we hypothesized that other factors, such as changes in mental state (i.e., stress, emotion, and cognitive load) and evoked potentials [i.e., heartbeat-evoked potential (HEP)], in addition to noise, could affect the ERP signals, leading to a decrease in performance (Zhang et al., 2020; Zheng et al., 2020). Some previous studies have sought to improve the classification performance by considering the changes in mental state (Ko et al., 2020; Zhang et al., 2020), but no study using HEP has been reported. Evoked potentials are difficult to remove or recover because, unlike noise, they do not cause a change in the dominant pattern of the EEG signal but are instead contained in the EEG signal itself (Schandry and Montoya, 1996; McCraty et al., 2009).

Heartbeat-evoked potentials are characteristic changes in brain waves caused by evoked potentials that can occur due to changes in cardiac activity, such as heart rhythms and heart rate variability (Schandry and Montoya, 1996; McCraty et al., 2009; Park et al., 2015). The vagus nerve transmits cardiac output information *via* the visceral-afferent pathway (medulla, amygdalae, hypothalamic and thalamic nuclei, and nucleus tractus solitarius) from the heart to the brain (Montoya et al., 1993; Janig, 1996; Nieuwenhuys et al., 2007). HEPs reflect a synchronization in the communication between the brain and heart based on efferent and afferent pathways, leading to evoked potentials involving changes in alpha activation in EEG signals (McCraty et al., 2009; Park et al., 2015; Park and Whang, 2018). HEP is divided into two components. The first HEP component (50–250ms post-R-wave) is defined as the interval required for afferent information from the heart to reach the brain. An increase in afferent processing is indicated by the synchronization of the alpha wave. The second HEP component (250–600ms post-R-wave) is defined as the time interval needed for blood pressure to reach the brain area from the heart. When the blood pressure wave synchronizes with brain activity, alpha synchronization occurs, which is associated with the higher cognitive centers’ processing of the sensory input (Wölk et al., 1989; McCraty et al., 2009; Park et al., 2015).

As previously mentioned, HEP causes evoked potentials in EEG signals due to the synchronization between the brain and heart. HEP is similar to ERP in terms of how it causes evoked potentials in EEG signals based on a trigger point. We believe that HEP can be contained in the ERP when there is little difference in the trigger time between the heartbeat and the event. In ERP analyses, the detection of a significant ERP pattern may be impeded by evoked potentials overlapping a heartbeat. Thus, this study sought to determine the effect of evoked potentials by HEPs on changes in significant patterns in ERP signals during a cognitive task, as well as the ERP-based classification performance of mental workload, by separating ERP trials that are affected by HEP from those that are not.

## Materials and Methods

### Participants

Fourteen undergraduate students (seven male and seven female) aged between 22 and 29years (mean 25.2±3.4) participated in the experiment. Each subject participated voluntarily and was paid 100,000 KRW. All participants were right-handed and had no family or medical history of cardiovascular, autonomic, or central nervous system disorders. All participants were required to abstain from alcohol, cigarettes, and caffeine for 24h prior to the experiment and to sleep normally. Informed consent was acquired from all participants who were notified of the restrictions and requirements. All experimental protocols were approved by the Sangmyung University Institutional Bioethics Review Board in Seoul, South Korea (BE2019-46).

### Experimental Stimuli and Apparatus

#### ERP Task

**Figure 1:**
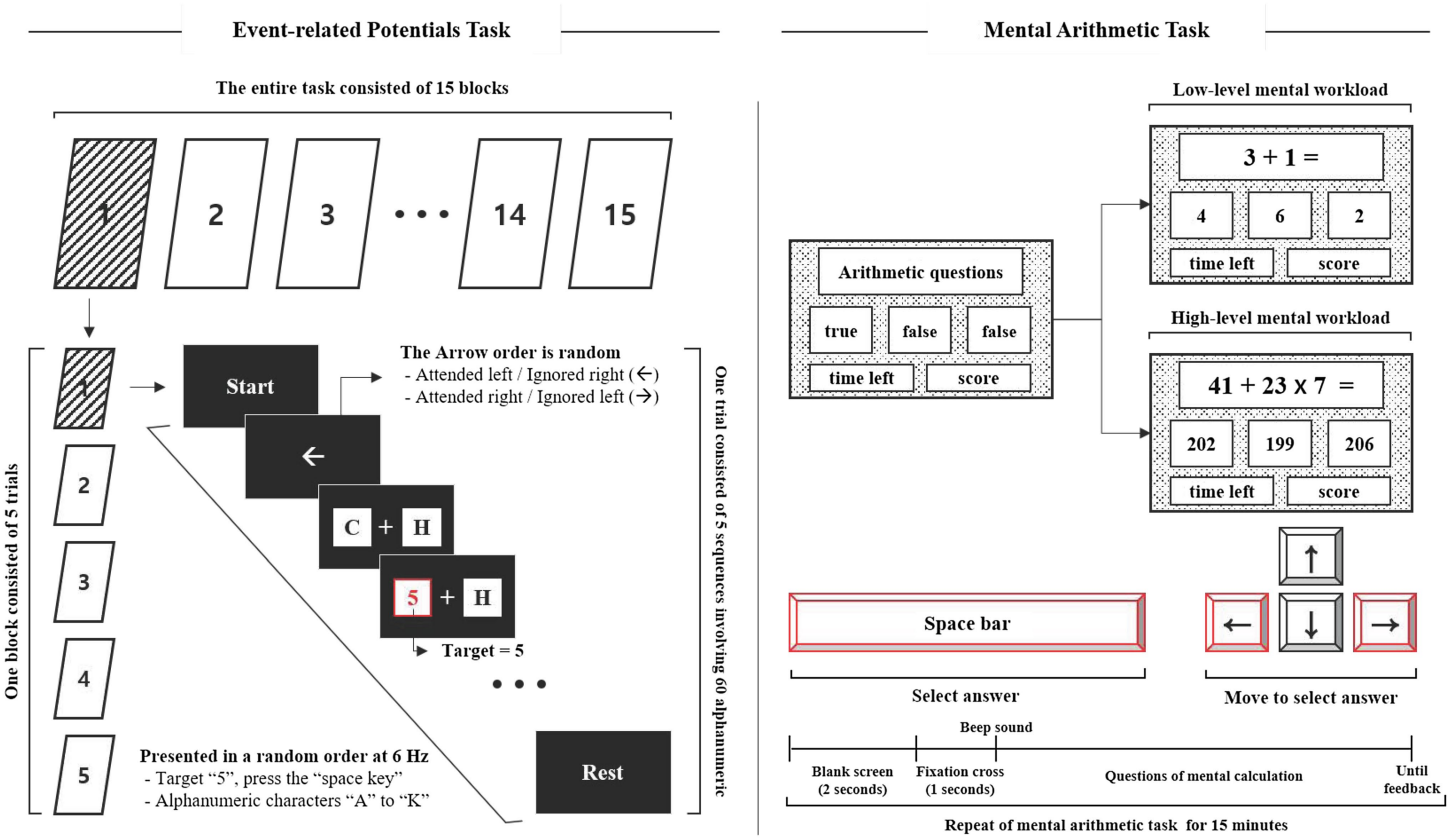
Experimental stimuli to measure ERP responses (left) and cause mental workload (right).

A stimulator was designed to measure the ERP response to mental workload based on our previous studies (Mun et al., 2012; Park et al., 2014, 2015, 2019). The stimulator was located on the left and right sides of the screen, and participants were required to focus their attention on the instructed side of the screen according to an arrow (to ignore the left side and attend to the right one, or vice versa). The stimulator consisted of the presentation of 12 alphanumeric characters involving non-targets (“A” to “K”) and a target (“5”). The alphanumeric characters were randomly updated at the rate of 6Hz. One trial consisted of five sequences involving 60 alphanumeric characters lasting 10s, with an inter-trial interval of 2s (total 60s). One block consisted of five trials, and the entire task consisted of 15 blocks. The target was presented with a probability of 5% within each trial, and the interval between targets lasted less than 1s to avoid overlapping ERPs during analysis, as shown in Figure 1.

#### Mental Workload: Mental Arithmetic Task

The mental arithmetic task was designed to cause mental workload (MWL) based on previous studies (So et al., 2017; Jost et al., 2019) and was divided into two task levels: low- and high-MWL. The low-MWL task consisted of easy questions involving single-digit addition and subtraction (i.e., 3+2, 4–1, with numbers ranging from 1 to 9). The high-MWL task consisted of difficult questions involving mixed arithmetic operations (i.e., 36×7–24, 43+72/9, number range 1–99). The mental arithmetic task questions were randomly presented within a defined range, and included a true answer result and two false confusion results. The two false results were automatically calculated by randomly adding or subtracting a number in the range of 1–5 from the correct answer. Participants were required to select the correct answer using the arrow and spacebar keys of the keyboard, as shown in Figure 1. ERP and mental arithmetic tasks were developed using LabVIEW2016 (National Instruments Inc., Austin, TX, United States).

### Experimental Procedure

**Figure 2:**
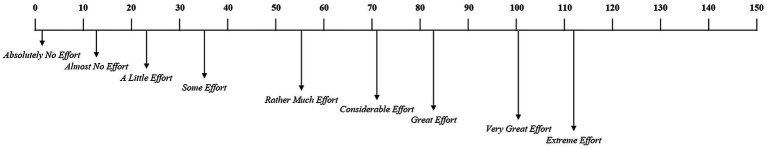
Subjective mental effort questionnaire (SMEQ).

**Figure 3:**
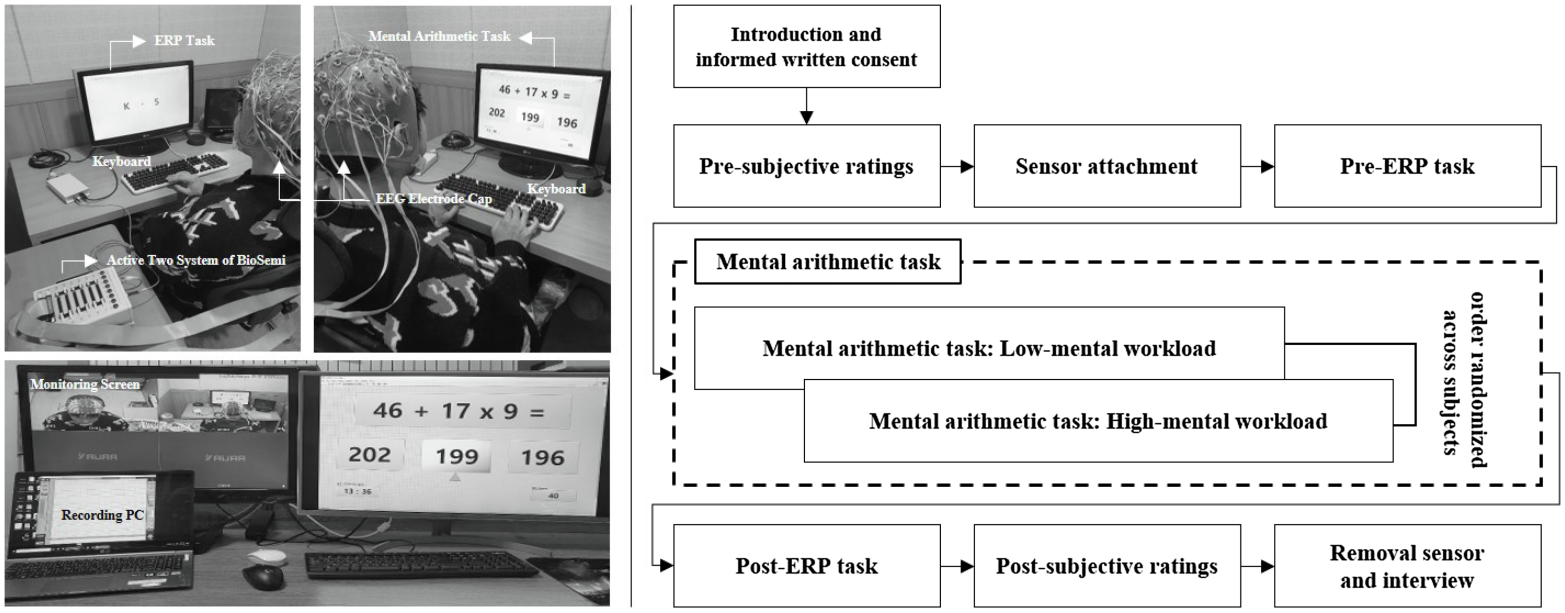
Experimental environment (left) and procedure (right).

Participants were required to report their MWL state as a subjective rating both before and after the experiment. The subjective mental effort questionnaire (SMEQ) (Sauro and Dumas, 2009), a questionnaire with a 0–150 scale for rating the MWL was used, as shown in Figure 2. Participants performed the pre-ERP task for 15min. Over the course of this session, all participants were required to fixate on a red cross at the center of the screen, 60cm from the display and press a spacebar key when presented with the target “5.” The performance and response times were measured for the target. Following the pre-ERP task, they performed the mental arithmetic task for 15min. All participants were asked to select the correct answer to the mental arithmetic question, from three options, using the arrow and spacebar keys of the keyboard. For each correct answer, the participant was awarded 10 points, whereas 10 points were deducted for an incorrect answer. In order to increase the subjects’ motivation and engagement, those who achieved the top 15% score were paid 150% of the test fee. Participants were divided into low-MWL and high-MWL task groups. On the first day, they performed either the low-MWL or High-MWL task and on the next day, they performed the other MWL task at the same time (e.g., first day low-MWL task and second day high-MWL task; the order randomized across subjects). Participants then performed the post-ERP task, which was the same as the pre-ERP task. The experimental environment and procedure are illustrated in Figure 3.

### Signal Processing and Data Acquisition

Electroencephalogram signals were recorded at a sampling rate of 2,048Hz from 64 channels mounted on an EEG electrode cap (Active-two, Biosemi S.V., Amsterdam, Netherlands) based on the international 10–20 montage with separate reference and ground electrodes for each system (common mode sense, CMS and driven right leg, DRL, respectively). Impedance from all electrodes was kept below 5kΩ (below 10kΩ for the two eye channels). The measured EEG signals were down-sampled to 512Hz and re-referenced using a common average referencing (CAR) procedure (Perrin et al., 1989). The CAR was calculated by subtracting the average potential over all the channels from each channel. This re-referencing maintains the activity from local sources while removing the global background activity (Lew et al., 2012). To avoid contaminating meaningful ERP patterns, we minimized preprocessing by specifying a threshold for each trial. In the trials, where the amplitude exceeded ±100μV at any electrode, we conducted an independent component analysis to remove oculomotor and muscle artifacts by visual inspection. Additionally, a few trials with anomalous patterns were excluded due to the difficulty of reconstruction (Rampone et al., 2019; Katus et al., 2020; Li et al., 2020).

The ERP analysis procedure was as follows. (1) The EEG signals were divided into epochs of 1,000ms based on the event (200ms before target onset to 800ms after target onset) and averaged. (2) Average ERPs were constructed from trials containing 1,000ms epochs of raw data, of which the pre-stimulus period (first 200ms) was used to correct the baseline averaged ERP epochs lasting 800ms (Mitchell et al., 2016). (3) Next, the P600 latency and amplitude were detected from the averaged ERP epochs. The P600 latency and amplitude were determined from its highest point and mean amplitude, respectively, within a time window between 530 and 750ms following stimulus presentation (Causse et al., 2016) at the F3, F4, C3, C4, P3, P4, O1, and O2 electrodes (Mun et al., 2014; Park et al., 2015, 2019). Each electrode site corresponds to a brain region identified by a letter: frontal (F), central (C), parietal (P), and occipital (O). These brain areas are associated with the following functions: (1) the frontal area is associated with reasoning, motor skills, higher level cognition, and expressive language; (2) the central area is associated with motor and sensory information; (3) the parietal area is associated with processing of tactile sensations; and (4) the occipital area is associated with interpreting visual stimuli and information (Dimond and Beaumont, 1974). All signal processing and data analyses were performed using EEGlab, a MATLAB toolbox (2020b, Mathworks Inc., Natick, MA, United States).

**Figure 4:**
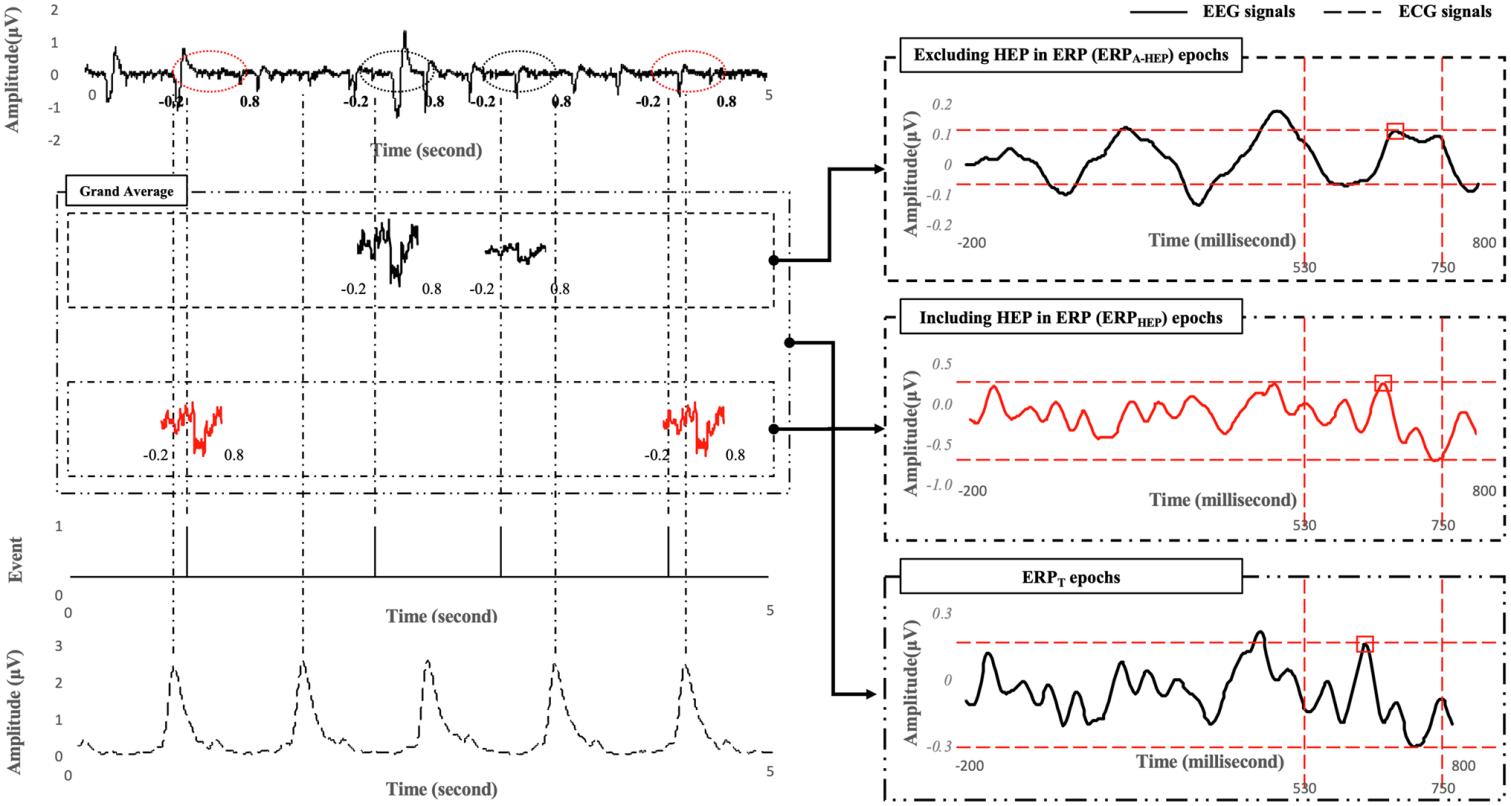
Examples of signal processing compared across ERPA-HEP, ERPHEP, and ERPT.

Participants were required to perform all the 375 trials for the ERP task. In order to assess the effect of the evoked potentials caused by heartbeat (i.e., the alpha activation of HEPs) on the latency and amplitude of the P600 component in ERPs, and the classification performance in distinguishing between low- and high-MWL states, the entire ERP epochs were categorized into two conditions based on the heartbeat within each ERP epoch: including the heartbeat within the ERP epoch (denoted ERP_HEP_) or not including the heartbeat within the ERP epoch (denoted ERP_A-HEP_, for anti-HEP). ERP_HEP_ and ERP_A-HEP_ accounted for 171.79±10.31 and 203.21±10.31 trials, respectively, on average, for each subject. More specifically, ERP_HEP_ was defined as containing the heartbeat during the period from 280 to 700ms in the ERP epochs after the target was presented. This period was determined by considering the interval in which the evoked potentials overlapped the heartbeat (50–250ms after the heartbeat) and events related to P600 (530–750ms after target). ERP_A-HEP_ was defined as not including the heartbeat within the same period. Finally, we defined the ERP_T_ condition as including all the ERP_A-HEP_ and ERP_HEP_ trials. We divided the ERP_A-HEP_, ERP_HEP_, and ERP_T_ trials from entire ERP epochs, and compared their classification performance in separating low- and high-MWL states and the pattern of latency and amplitude of the P600 component, as shown in Figure 4.

### Statistical Analysis and Classification

This study followed a within-subject design with respect to low- and high-MWL. A paired *t*-test was used based on the results of the Shapiro–Wilk normality test. Differences between pre- and post-features were calculated to take into account the state before the task. The confidence level in the statistical tests was controlled by the number of individual hypotheses (i.e., *α*=05∕n) based on the Bonferroni correction. Bonferroni correction was performed to assess statistical significance while correcting for multiple comparisons (Dunnett, 1955; Johnson et al., 2019). Thus, the statistically significant levels of performance and ERP measures were set to 0.025 (accuracy and response time, *α*=0.05/2=0.025) and 0.0031 (ERP latency and amplitude at eight electrodes, *α*=0.05/16=0.0031). The effect size based on Hedges’ *g* was calculated to assess not only the statistical significance, but also the practical significance (Hedges and Olkin, 2014). All effect sizes were corrected for small sample sizes according to Hedges’ *g*. We also calculated the expected effect size for the paired t-test (Hedges’ *g*) using G*power software (Faul et al., 2007). The expected effect size in this study was 0.781 (paired *t*-test).

In EEG research, the radial basis function kernel-based support vector machine (RBF-SVM) is considered one of the optimized classifiers (Alomari et al., 2013). We used RBF-SVM to conduct a binary classification for a total of 28 samples × number of features after feature selection and standardization. The optimized kernel scales for each condition were as follows: ERP_T_, 117.8; ERP_HEP_, 2.7; and ERP_A-HEP_, 21.5. We conducted 10-fold cross-validation and represented the performance of the classification using accuracy, sensitivity, specificity, and area under the curve (AUC) for the receiver operating characteristics curve. Statistical analysis and classification were performed using the Statistics and Machine Learning Toolbox in MATLAB (2020b, Mathworks Inc., Natick, MA, United States).

## Results

### Subjective Ratings: SMEQ

**Figure 5:**
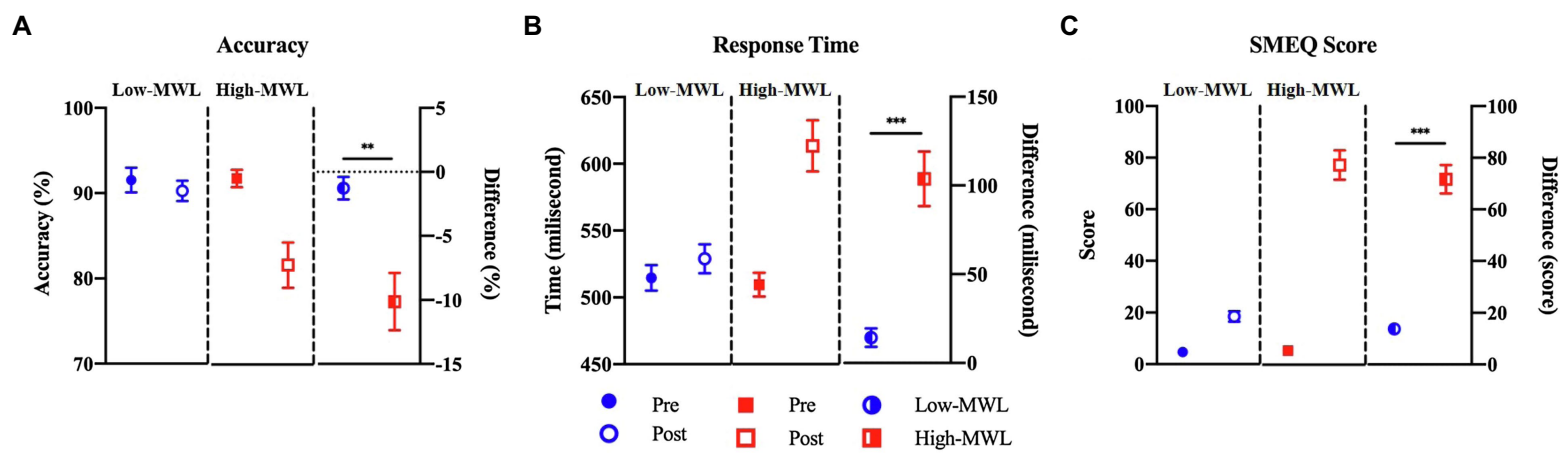
Comparison of accuracy **(A)**, response time **(B)** for target in ERP task, and SMEQ score **(C)** between the low- and high-MWL conditions (paired *t*-test; ^**^p<0.0031 and ^***^p<0.001).

Figure 5 represents the comparison of the SMEQ scores between low- and high-MWL conditions. In the paired t-test, the SMEQ score of the high-MWL condition was significantly higher than that of the low-MWL condition [*t*(13)=−9.238, Hedges’ *g*=3.796, 95% CI 2.556–5.036, *p*<0.001]. The mean (*M*) and standard deviation (*SD*) for each condition were as follows: low-MWL, *M*=13.79 and *SD*=6.37; high-MWL, *M*=71.64 and *SD*=20.59. Hedges’ *g* satisfied the expected effect size for the paired t-test in this study.

### ERP Task Performance: Accuracy and Response Time

A paired *t*-test of target accuracy in the ERP task showed a significant difference between the low- (*M*=−1.27, *SD*=3.29) and high-MWL (*M*=−10.13, *SD*=8.38) conditions [*t*(13)=3.762, Hedges’ *g*=−1.392, 95% CI –2.217 to −0.566, *p*<0.01]. The response time of the high-MWL (*M*=14.30, *SD*=19.54) condition was significantly delayed compared to the low-MWL (*M*=103.67, *SD*=57.76) condition [*t*(13)=−4.643, Hedges’ *g*=2.073, 95% CI 1.154 to −2.991, *p*<0.001], as shown in Figure 5. Hedges’ *g* for the performance of the ERP task satisfied the expected effect size for the paired t-test in this study.

### ERP Waveform: P600 Amplitude and Latency

#### P600 Amplitude

**Figure 6:**
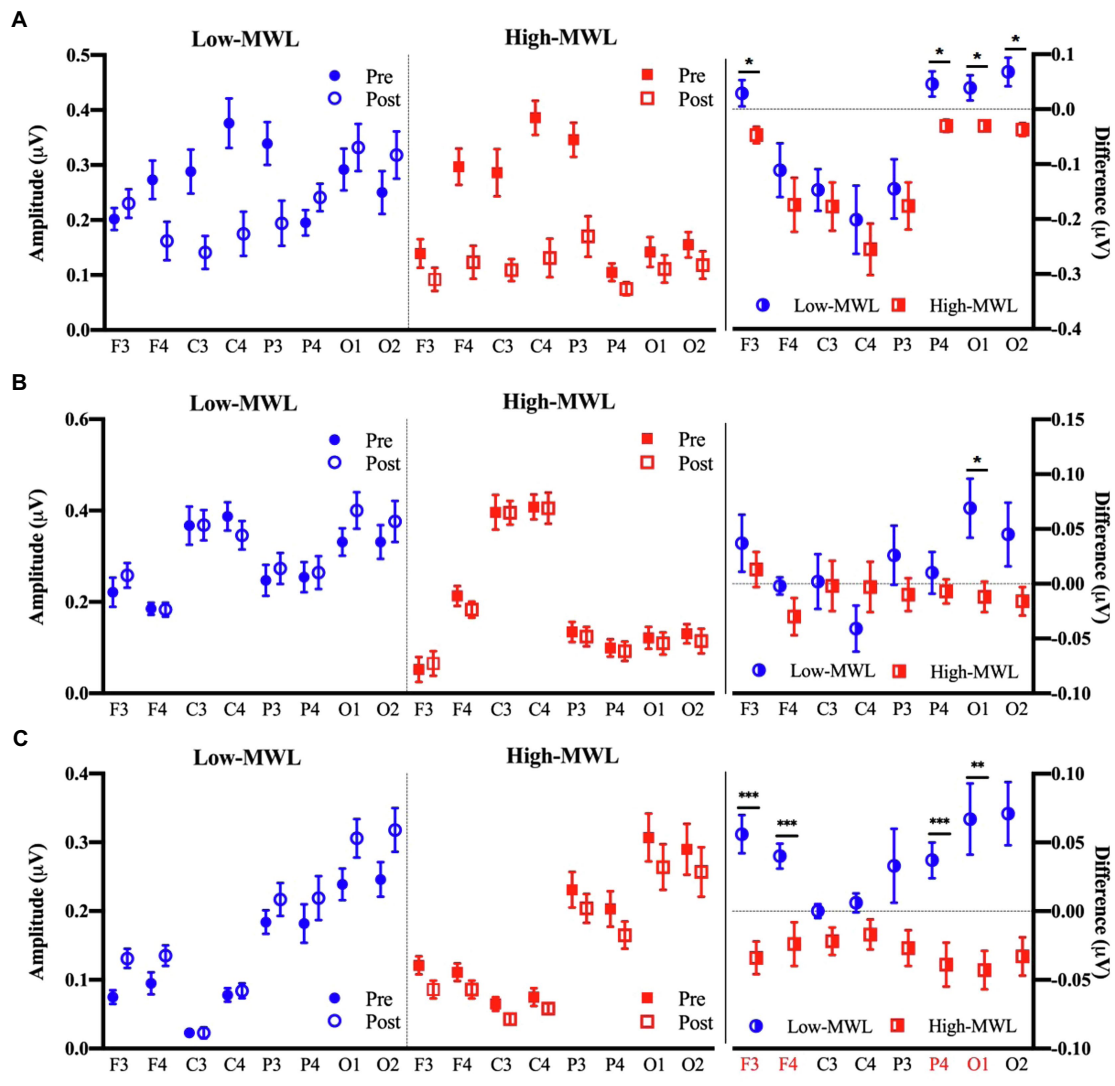
Comparison of the P600 amplitude in ERP between the low- and high-MWL conditions. **(A)** ERPT condition. **(B)** ERPHEP condition. **(C)** ERPA-HEP condition (paired *t*-test; ^*^p<0.05, ^**^p<0.0031, and ^***^p<0.001).

Figure 6 represents the results of the statistical analysis of the P600 amplitudes, comparing the low- and high-MWL conditions, for the ERP_T_, ERP_A-HEP_, and ERP_HEP_ epochs. In the cases of ERP_T_ and ERP_HEP_, no significant differences between low- and high-MWL conditions were found at any electrode site (F3, F4, C3, C4, P3, P4, O1, and O2) after Bonferroni correction (*p*>0.0031). However, in the case of ERP_A-HEP_, a paired t-test revealed a significant difference between the P600 amplitudes of the low- and high-MWL conditions at F3 [*t*(13)=5.505, Hedges’ *g*=−1.988, 95% CI –2.893 to −1.082, *p*<0.001], F4 [*t*(13)=4.787, Hedges’ *g*=−1.265, 95% CI –2.076 to −0.453, *p*<0.001], P4 [*t*(13)=4.383, Hedges’ *g*=−1.559, 95% CI –2.281 to −0.616, *p*<0.001), and O1 [*t*(13)=3.818, Hedges’ *g*=−1.391, 95% CI –2.217 to −0.566, *p*<0.0031]. No significant differences were found at the other electrode sites (C3, C4, P3, and O2). Detailed results are presented in Table 1. Hedges’ *g* for the P600 amplitude satisfied the expected effect size for the paired t-test.

**Table 1:** Comparison by paired *t*-test of the P600 amplitude between low- and high-MWL conditions.

	Site	Condition	N	Mean	SD	t	p	Effect size	
								Hedges’ g	95% CI
*ERP_T_*	*F_3_*	Low-MWL	14	0.03	0.09	2.865	0.0133 (>0.05)	−1.046	−1.836~−0.256
		High-MWL	14	−0.05	0.06				
	*P_4_*	Low-MWL	14	0.05	0.09	2.784	0.0155 (>0.05)	−1.149	−1.948~−0.349
		High-MWL	14	−0.03	0.04				
	*O_1_*	Low-MWL	14	0.04	0.09	2.556	0.0239 (>0.05)	−1.043	−1.833~−0.254
		High-MWL	14	−0.03	0.03				
	*O_2_*	Low-MWL	14	0.07	0.10	3.453	0.0043 (>0.05)	−1.444	−2.276~−0.613
		High-MWL	14	−0.04	0.04				
*ERP_HEP_*	*O_1_*	Low-MWL	14	0.07	0.10	2.658	0.0197 (>0.05)	−1.012	−1.799~−0.225
		High-MWL	14	−0.01	0.05				
*ERP_A-HEP_*	*F_3_*	Low-MWL	14	0.06	0.05	5.505	0.0001 (>0.001)	−1.988	−2.893~−1.082
		High-MWL	14	−0.03	0.04				
	*F_4_*	Low-MWL	14	0.04	0.03	4.787	0.0004 (>0.001)	−1.265	−2.076~−0.453
		High-MWL	14	−0.02	0.06				
	*P_4_*	Low-MWL	14	0.04	0.05	4.383	0.0007 (>0.001)	−1.449	−2.281~−0.616
		High-MWL	14	−0.04	0.06				
	*O_1_*	Low-MWL	14	0.07	0.10	3.818	0.0021 (>0.001)	−1.391	−2.217~−0.566
		High-MWL	14	−0.04	0.05				
	*O_2_*	Low-MWL	14	0.07	0.08	3.298	0.0058 (>0.05)	−1.499	−2.337~−0.661
		High-MWL	14	−0.03	0.05				

*Results are not shown when the p value is greater than 0.05*.

#### P600 Latency

**Figure 7:**
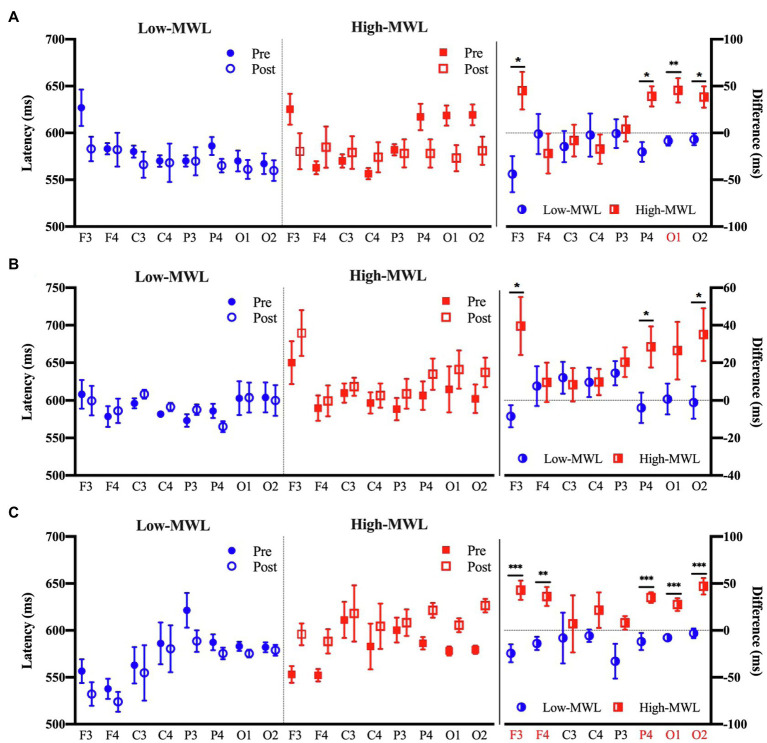
Comparison of the P600 latency in ERP between the low- and high-MWL conditions. **(A)** ERPT condition. **(B)** ERPHEP condition. **(C)** ERPA-HEP condition (paired t-test; ^*^p<0.05, ^**^p<0.0031, and ^***^p<0.001).

Figure 7 represents the results of the statistical analysis comparing the P600 latency between the low- and high-MWL conditions for the ERP_T_, ERP_A-HEP_, and ERP_HEP_ epochs. In the case of ERP_T_, a paired t-test revealed a significant difference between low- and high-MWL conditions at the O1 site only [*t*(13)=−3.935, Hedges’ *g*=1.462, 95% CI 0.628 to 2.296, *p*<0.0031], while no significant differences were found at the other electrode sites. In the case of ERP_HEP_, no significant differences were observed between the P600 latencies of low- and high-MWL conditions at any electrode site after Bonferroni correction (*p*>0.0031). For the ERP_A-HEP_, P600 latency was significantly prolonged in the high-MWL condition compared with the low-MWL condition at F3 [*t*(13)=−4.348, Hedges’ *g*=1.823, 95% CI 0.942 to 2.704, *p*<0.001], F4 [*t*(13)=−3.833, Hedges’ *g*=1.533, 95% CI 0.690 to 2.375, *p*<0.001], P4 [*t*(13)=−4.283, Hedges’ *g*=1.662, 95% CI 0.803 to 2.521, *p*<0.001], O1 [*t*(13)=−5.115, Hedges’ *g*=1.714, 95% CI 0.848 to 2.581, *p*<0.001], and O2 [*t*(13)=−5.526, Hedges’ *g*=1.871, 95% CI 0.983 to 2.760, *p*<0.001] sites. No significant differences were found at the other electrode sites (C3, C4, and P3). Detailed results are presented in Table 2. Hedges’ *g* for the P600 latency satisfied the expected effect size for the paired t-test.

**Table 2:** Comparison by paired t-test of the P600 latency the low- and high-MWL conditions.

	Site	Condition	N	Mean	SD	t	p	Effect Size	
								Hedges’ g	95% CI
ERP_T_	F_3_	Low-MWL	14	−44.00	72.29	−3.023	0.0098 (>0.05)	1.208	0.403~2.014
		High-MWL	14	45.00	75.02				
	P_4_	Low-MWL	14	−20.29	39.21	−3.284	0.0059 (>0.05)	1.493	0.655~2.331
		High-MWL	14	39.00	40.20				
	O_1_	Low-MWL	14	−8.57	18.89	−3.935	0.0017 (>0.0031)	1.462	0.628~2.296
		High-MWL	14	45.43	48.71				
	O_2_	Low-MWL	14	−6.86	23.60	−3.067	0.0090 (>0.05)	0.750	−0.016~1.516
		High-MWL	14	38.29	42.81				
ERP_HEP_	F_3_	Low-MWL	14	−8.43	21.95	−2.425	0.0306 (>0.05)	1.098	0.303~1.893
		High-MWL	14	39.57	57.79				
	P_4_	Low-MWL	14	−4.00	30.09	−3.500	0.0039 (>0.05)	0.903	0.125~1.680
		High-MWL	14	28.43	40.93				
	O_2_	Low-MWL	14	−1.14	31.95	−2.423	0.0308 (>0.05)	0.830	0.058~1.602
		High-MWL	14	35.00	52.62				
ERP_A-HEP_	F_3_	Low-MWL	14	−24.43	35.54	−4.348	0.0008 (>0.001)	1.823	0.942~2.704
		High-MWL	14	42.86	38.24				
	F_4_	Low-MWL	14	−13.86	26.57	−3.833	0.0021 (>0.001)	1.533	0.690~2.375
		High-MWL	14	36.14	37.71				
	P_4_	Low-MWL	14	−11.86	33.92	−4.283	0.0009 (>0.001)	1.662	0.803~2.521
		High-MWL	14	35.14	21.18				
	O_1_	Low-MWL	14	−7.71	14.42	−5.115	0.0002 (>0.001)	1.714	0.848~2.581
		High-MWL	14	27.57	25.28				
	O_2_	Low-MWL	14	−3.14	19.25	−5.526	0.0001 (>0.001)	1.871	0.983~2.760
		High-MWL	14	47.00	32.64				

*Results are not represented when the value of p is greater than 0.05*.

**Figure 8:**
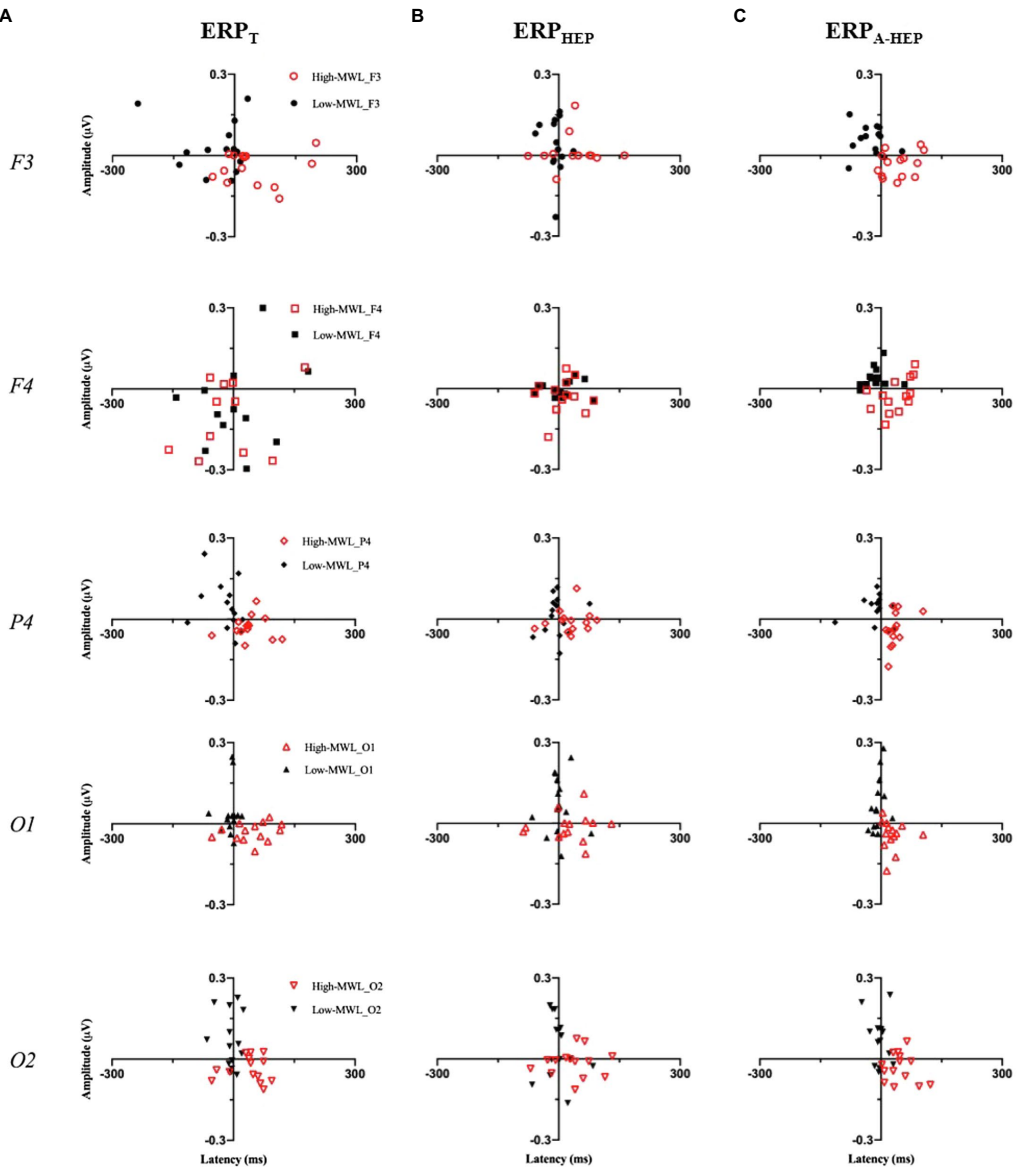
Scatterplots of P600 amplitude (X axis) and latency (Y axis) between low- and high-MWL for the ERPT, ERPHEP, and ERPA-HEP conditions.

To visually confirm the pattern of ERP features between low- and high-MWL, we produced scatterplots with amplitude and latency as the two axes for the ERP_T_, ERP_HEP_, and ERP_A-HEP_ conditions. As shown in Figure 8, only the ERP_A-HEP_ condition revealed a clear pattern distinguishing the two MWL states. A paired t-test of the heart rate showed no significant difference between the low-MWL (0.81±0.05) and high-MWL (0.79±0.05) conditions [*t*(13)=1.479, *p* =0.163].

### Classification and Correlation Results

#### Classification

**Figure 9:**
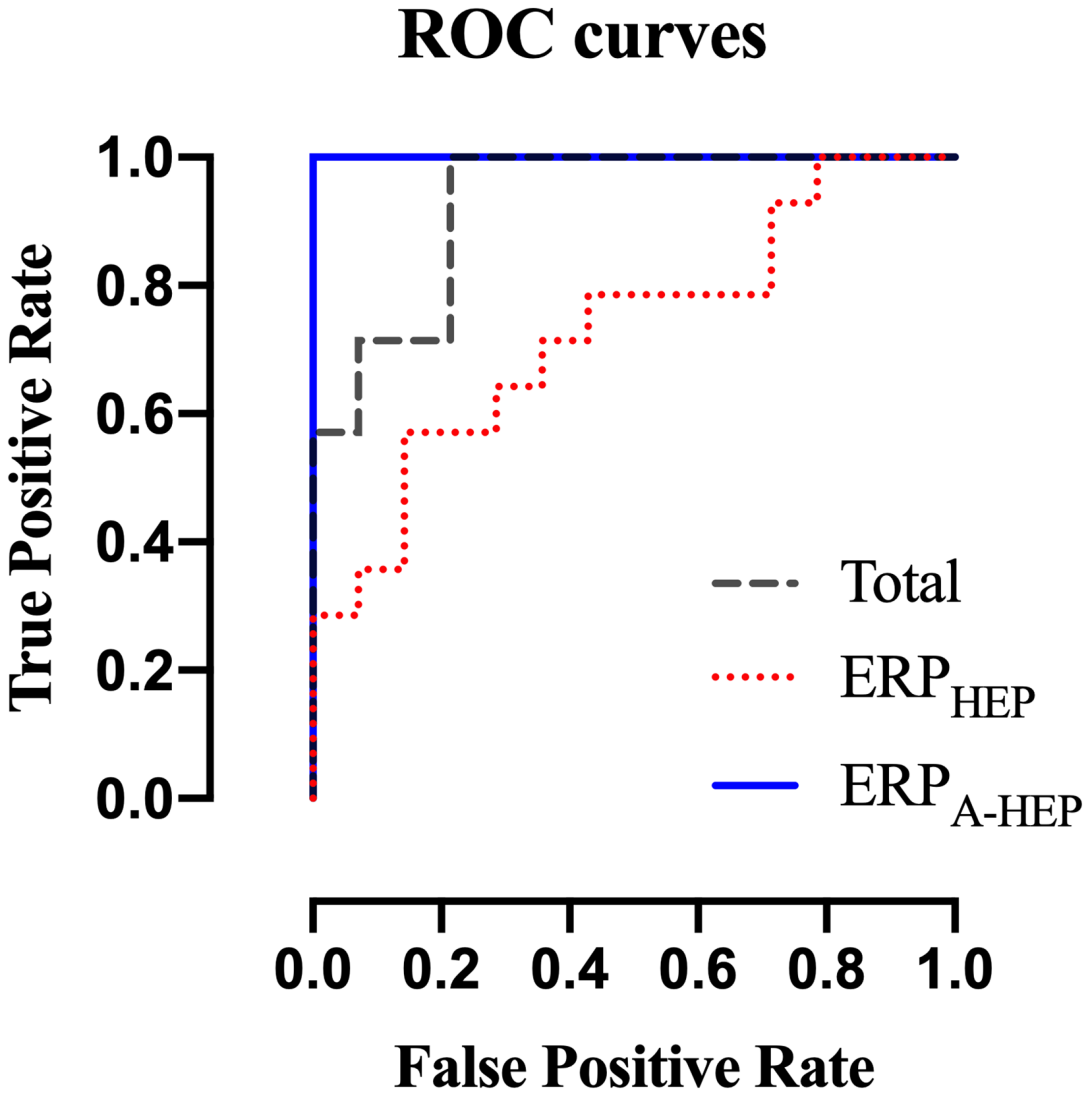
Receiver operating characteristics curves for ERPT, ERPHEP, and ERPA-HEP according to the RBF-SVM classifier.

We compared the classification performance of ERP_T_, ERP_HEP_, and ERP_A-HEP_ in distinguishing between low- and high-MWL in order to assess the effect of the HEP on the predictive power of ERPs (10-fold cross-validation). The RBF-SVM was selected as the classification method since it is widely used in EEG-related studies. In the three conditions (ERP_T_, ERP_HEP_, and ERP_A-HEP_), for all ERP amplitude and latency, the following values of the performance metrics were achieved as: accuracy: 85.7,% 71.4, and 100%, respectively; sensitivity, 85.7, 64.3, and 100%; specificity, 85.7, 78.6, and 100%; and AUC: 0.93, 0.76, and 1, as shown in Table 3 and Figure 9.

**Table 3:** Results of the classification by RBF-SVM (10-fold cross-validation) with ERP_T_, ERP_HEP_, and ERP_A-HEP_ epochs (*n*=24).

	Condition	Accuracy (%)	Sensitivity (%)	Specificity (%)	AUC
RBF-SVM (10-fold cross-validation)	ERP_T_	85.7	85.7	85.7	0.93
	ERP_HEP_	71.4	64.3	78.6	0.76
	ERP_A-HEP_	100	100	100	1

#### Correlation

We conducted a correlation analysis among ERP features and SMEQ scores and compared the correlation coefficients for the ERP_T_, ERP_HEP_, and ERP_A-HEP_ conditions. Specifically, we assessed the partial correlation between post-ERP features and post-SMEQ scores while controlling for two covariates (pre-ERP features and pre-SMEQ scores) to take into account the state before the experiment (Liu, 1988). In the ERP_T_, ERP_HEP_, and ERP_A-HEP_ conditions, we found significant correlations between SMEQ score and seven, four, and ten ERP features, respectively. The correlation coefficients in the ERP_A-HEP_ condition (amplitude: 0.410 to 0.885; latency: −0.401 to −0.586) were higher than those of the ERP_T_ (amplitude: 0.414 to 0.497; latency: −0.432 to −0.483) and ERP_HEP_ (amplitude: 0.440; latency: −0.419 to −0.493) conditions. Detailed correlation results are presented in Table 4.

**Table 4:** Partial correlation analysis between ERP features and SMEQ scores for each condition (ERP_T_, ERP_HEP_, and ERP_A-HEP_).

		F_3_	F_4_	C_3_	C_4_	P_3_	P_4_	O_1_	O_2_
**P600 Latency in ERP**									
SMEQ score	ERP_T_	−0.473 (*p* >0.05)	–	–	–	–	−0.432 (*p* >0.05)	−0.483 (*p* >0.05)	−0.440 (*p* >0.05)
	ERP_HEP_	−0.440 (*p* >0.05)	–	–	–	–	−0.493 (*p* >0.05)	–	−0.419 (*p* >0.05)
	ERP_A-HEP_	−0.401 (*p* >0.05)	–	–	–	–	−0.474 (*p* >0.05)	−0.496 (*p* >0.05)	−0.586 (*p* >0.01)
**P600 Amplitude in ERP**									
SMEQ score	ERP_T_	0.461 (*p* >0.05)	–	–	–	–	0.414 (*p* >0.05)	–	0.497 (*p* >0.01)
	ERP_HEP_	–	–	–	–	–	0.440 (*p* >0.05)	–	–
	ERP_A-HEP_	**0.885** **(*p* >0.001)**	**0.783** **(*p* >0.001)**	–	–	0.410 (*p* >0.05)	**0.612** **(*p* >0.001)**	0.600 (*p* >0.01)	**0.632** **(*p* >0.001)**

*Results are not represented when the p value is greater than 0.05 (light gray, p<0.05; dark gray, p<0.01; and bold black, p<0.001)*.

## Discussion and Conclusion

This study sought to determine the effect of HEP on the amplitude and latency of the P600 component of ERPs and to compare the classification accuracy for the MWL task among the ERP_T_, ERP_HEP_, and ERP_A-HEP_ conditions. SMEQ score and MWL performance (accuracy and response time for target) were significantly different between low- and high-MWL tasks. These results confirmed that a difference in the MWL state of participants resulted from low- and high-mental arithmetic tasks. The P600 ERP amplitude and latency were significantly lower and higher, respectively, in the high-MWL than in the low-MWL tasks. This result is consistent with that of previous studies (Baetens et al., 2011; Mun et al., 2012, 2017; Park et al., 2015, 2019). Late positive potentials (LPPs), such as the P600 and P700 components reflect a high-level MWL required to process difficult tasks (Li et al., 2008; Pastor et al., 2008; Mun et al., 2012, 2014). LPPs are seen as a delayed P300 component and are related to high-level MWL (Friederici et al., 1993; Mun et al., 2012). Reduced LPP amplitudes reflect a decrease in cognitive and neural resources caused by MWL, impairing the attentional allocation mechanisms (Kato et al., 2009; Mun et al., 2012, 2014).

The analysis revealed significant differences in the P600 amplitude and latency among the ERP_T_, ERP_HEP_, and ERP_A-HEP_ conditions. The number of features (i.e., P600 amplitude and latency in each brain region) with significant differences (*p* <0.0031) between low- and high-MWL was greater in the ERP_A-HEP_ (10 significant features) than in the other conditions (ERP_T_, one significant feature; ERP_HEP_, no significant feature). The P600 amplitude and latency in the ERP_A-HEP_ condition revealed a stronger correlation with SMEQ scores than the other two conditions. These results suggest that the pattern of P600 amplitude and latency in the ERP_A-HEP_ condition, not affected by the heartbeat, revealed a clearer response to ERP stimuli, without interference from other evoked potentials, compared with other conditions that were affected by the heartbeat.

The vagus nervous system in the heart continuously communicates with the brain *via* efferent and afferent pathways. Neuronal connectivity causes an evoked potential (i.e., alpha rhythms) in brain waves, known as the HEP (Schandry and Montoya, 1996; McCraty et al., 2009; Park et al., 2015). Many previous studies have used the HEP phenomenon in various research fields, such as attention (Petzschner et al., 2019), anxiety (Judah et al., 2018; Pang et al., 2019), mental workload (Park et al., 2015), emotion (Couto et al., 2015), sleep (Lechinger et al., 2015), and medicine (Leopold and Schandry, 2001; Perogamvros et al., 2019; Flasbeck et al., 2020).

We divided the ERP trials into three conditions according to whether the heartbeat had an effect on the evoked potential on EEG oscillations. The condition related to the heartbeat (ERP_HEP_) resulted in a change in the P600 amplitude for the target due to the overlap with the evoked potentials induced by the heartbeat, which also led to variations in latency. The phenomenon whereby the evoked potentials caused by the heartbeat affect EEG oscillations has been reported in many previous studies (Wölk et al., 1989; Schandry and Montoya, 1996; McCraty et al., 2009; Park et al., 2015, 2019; Villena-Gonzalez et al., 2017; Perogamvros et al., 2019). Our argument regarding the effect of cardiac activity on the P600 in ERP is supported by the high statistical significance and correlation coefficient we observed in the ERP_A-HEP_ condition compared with the ERP_HEP_ one.

This study also assessed the classification performance using P600 features between low- and high-MWL for the three conditions (ERP_T_, ERP_HEP_, and ERP_A-HEP_), finding that the accuracy in the ERP_A-HEP_ condition increased by 14.3 and 28.6% compared to the ERP_T_ and ERP_HEP_ conditions, respectively. No previous studies have tried to detect the pure ERP components and improve the MWL classification performance based on ERP while considering the effect of the evoked potentials (i.e., HEP) caused by the cardiac activity (i.e., heartbeat). However, some studies have attempted to improve the accuracy of brain activity classification using approaches similar to that used in our studies. Brain oscillations can be affected by various mental states, such as fatigue, stress, emotion, and mental workload. Changes in mental state can affect EEG oscillations and interfere with the targeting response. This leads to a decrease in classification performance (Lorenz et al., 2014; Myrden and Chau, 2017; Zhang et al., 2020; Zheng et al., 2020). A previous study compared brain-computer interface (BCI) performance under low- and high-stress conditions and reported that the use of steady-state visually evoked potential-based BCI under stress leads to a decrease in accuracy and an increase in the required concentration and the resulting fatigue (Zhang et al., 2020). Another study revealed an increase in BCI performance considering the mental focus and lost-in-thought states of participants (Ko et al., 2020). In addition, many studies have sought to improve performance by considering various mental states (Myrden and Chau, 2017; Acı et al., 2019; Zhang et al., 2019; Foong et al., 2020), which is highly relevant to our research approach. Therefore, we believe that the evoked potential caused by the heartbeat must be considered to improve the detection of the evoked potential components in ERPs and that our approach can contribute to the measurement of accurate ERP responses and improve the performance in classifying MWL.

However, this study has several limitations. (1) Only the effects of the HEP on the P600 element of ERP were confirmed, and other components, such as P300, N400, and P200, were not considered. Since our approach selected trials suitable for each ERP element based on the heartbeat, other ERP components may behave similarly to the P600 component, although this needs to be confirmed through further research based on various task environments. (2) Our study sought to confirm a significant difference in MWL classification performance by ERP trials according to whether or not the HEP was affected. In future research, we will conduct a paradigm study that controls the timing of stimuli in ERP tasks based on heartbeats, or a method of training by classifying ERP trials based on whether they are affected by HEP.

In conclusion, this study confirmed that the performance in the classification of MWL states in the ERP_A-HEP_ condition was significantly superior to that of the ERP_T_ and the ERP_HEP_ conditions. We interpret these results as showing that the pattern of ERPs in the ERP_T_ and the ERP_HEP_ conditions, which were affected by the heartbeat, resulted from the overlap of the HEP and the ERPs. On the other hand, the pattern of ERPs in the ERP_A-HEP_ condition, which was not affected by the heartbeat, showed a clear or pure response to ERP stimuli without the effect of other evoked potentials. Therefore, in ERP studies, the effect of HEPs on ERP patterns (i.e., amplitude and latency in ERP components) needs to be considered in order to obtain a clear and pure ERP response. This study used the P600 component to improve ERP-based MWL classification performance, but the same approach can be used in the application of ERPs to various fields, such as brain-computer interface, emotion recognition, language processing, working memory, and neurotherapy.

## Data Availability Statement

The data generated and analyzed in this study are available from the corresponding author upon reasonable request.

## Ethics Statement

All experimental protocols were approved by the Sangmyung University Institutional Bioethics Review Board (SMUIBRB) in Seoul, South Korea (BE2019-46). The patients/participants provided their written informed consent to participate in this study.

## Author Contributions

SP: conceptualization, methodology, data analysis, experiment, and writing – original draft. JH: investigation, visualization, data analysis, and experiment. LK: conceptualization, writing – review and editing, and supervision. All authors contributed to the article and approved the submitted version.

## Funding

This work was partly supported by the Institute of Information & Communications Technology Planning & Evaluation (IITP) grant funded by the Korean Government (MSIT) (No. 2017-0-00432), for the development of a non-invasive integrated BCI SW platform to control home appliances and external devices by user’s thoughts *via* AR/VR interface.

## Conflict of Interest

The authors declare that the research was conducted in the absence of any commercial or financial relationships that could be construed as a potential conflict of interest.

## Publisher’s Note

All claims expressed in this article are solely those of the authors and do not necessarily represent those of their affiliated organizations, or those of the publisher, the editors and the reviewers. Any product that may be evaluated in this article, or claim that may be made by its manufacturer, is not guaranteed or endorsed by the publisher.

## Abbreviations

HEPs: heartbeat-evoked potentials
ERPs: event-related potentials
MWL: mental workload
ERP_HEP_: containing the heartbeat in ERP epochs
ERP_A-HEP_: not containing the heartbeat in ERP epochs
ERP_T_: ERP_A-HEP_ and ERP_HEP_ together
RBF-SVM: radial basis function-support vector machine
EEG: electroencephalogram
SMEQ: subjective mental effort questionnaire
CAR: common average referencing
ICA: independent component analysis
F: frontal region
C: central region
P: parietal region
O: occipital region
AUC: area under curve
ROC: receiver operating characteristics
LPPs: late positive potentials
BCI: brain-computer interface
SVEP: steady-state visually evoked potential
